# Experimental study and dynamic characteristics analysis of partially liquid-filled annulus tubes

**DOI:** 10.1371/journal.pone.0209011

**Published:** 2018-12-18

**Authors:** Hongyuan Sun, Ao Zhang, He Li

**Affiliations:** School of Mechanical Engineering and Automation, Northeastern University, Shenyang, Liaoning, China; University of Vigo, SPAIN

## Abstract

As a useful technology, the horizontal drilling for underground coalmine has been applied in many fields such as gas drainage, water control, exploration and accurate drilling borehole engineering. In this paper, the annulus tubes are used to simulate the fluid transportation process between drilling rod and casing tube when drilling horizontally. The main goal of this research is to analyze the effects of eccentricity, fluid velocity, outer tube length and volume fraction on dynamic characteristics of partially liquid-filled annulus tube in a drillstring system. According to the Euler-Bernoulli beam model, the partially liquid-filled inner tube model and outer tube model are established respectively when considering the effect of transport fluid inside the annulus tubes. Based on the vibration characteristics of the tube, the added mass coefficient is proposed to calculate the natural frequencies of the outer tubes. And natural frequency is the frequency at which a system tends to oscillate in the absence of any driving or damping force. According to the compared results of experiments and simulations, the natural characteristics of outer tube depend on the tube design parameter (outer tube length) and the volume fraction of liquid filled in the outer tube. Furthermore, experiments have verified that the natural frequencies of the outer tubes can be directly calculated from the length of outer tubes. Therefore, it is feasible to propose the method of attaching liquid mass to tube mass to obtain the natural frequencies of outer tubes. According to the results, it can be concluded that: (i) The natural frequencies of tube decrease as the length of the tube increases; (ii) The increase of volume fraction of liquid in the tube can significantly reduce the natural frequencies of tube; (iii) The fluid velocity and eccentricity have less effects on the natural frequencies of tube; (ⅳ) The added mass coefficient of outer tubes can be calculated and it is not a fixed value, it varies with the volume fraction of fluid in annulus tubes. In this work, the inner and outer tube models are established respectively for fluid transportation process between a horizontal drilling rod and casing tube. The effects of four factors on the natural frequencies are compared, and the concept of added mass coefficient is proposed to obtain reasonable tube natural frequencies.

## Introduction

The losses caused by tube vibration in a drilling system amounted to almost tens of billions of dollars each year in advanced industrialized countries. The sudden change of load and strong vibration are inevitable in the process of mechanical work, which will cause strong disturbance to the transmission tubes and lead to instable work situation [1]. In severe cases, the tubes may be damaged and more incalculable losses may be caused. Therefore, it is very important to study the vibration of tube transportation during drilling.

The basic vibration of outside machine and the resonance of tube are the most important factors that intensify the vibration of the annulus tube. At the same time, the drilling rig can generate foundation vibration during drilling operation, which is unavoidable. Therefore, it is necessary to reduce the vibration of the annulus tube so as to avoid the resonance. In the process of drilling, with the deepening of the length of drill string in coal seam, the natural frequencies of the tube cannot be directly tested. When the level of liquid is different, the vibration characteristics of the annulus tubes can also be affected. Undoubtedly this problem leads to the increasing difficulty of the fluid transportation. The research on the dynamic characteristics of the annulus tubes is very necessary.

The interests in partially liquid-filled tubes of the drillstring system started many years ago. Ozbelge and Beyaz discussed the solid–liquid mixture flow pressure drop and the effect of the local solid concentrations on radial solid concentration profiles [2]. Ergin and Temarel studied the dynamic characteristics of a partially-filled, horizontal, cylindrical shell [3]. Han examined solid–liquid mixture upward hydraulic transport of solid particles in vertical and inclined annuli with a rotating inner cylinder. According to their study, the use of an average particle rise velocity to evaluate particle transport performance in an inclined annulus would be misleading which leading to high effective fluid and particle velocities [4]. Torre proposed the method of added mass effects to investigate the natural frequencies of the fluid–structure system [5]. Behnam Amanna investigated two-phase liquid solid flow in the annular space of a drilling well, he concluded that due to greater drag forces applied to cuttings, transportation of cuttings would be easier at higher drill tube speeds [6]. Hidalgo presented an experimental and numerical study on the flexural vibration of horizontal circular tubes partially filled with liquid, a method of added mass of liquid was used. However, only different amounts of filling liquid and volume of fraction were considered in the experiment [7].

Despite all these theoretical basis and experimental works, the study on experiment of tube’s natural vibration frequencies with partially liquid filled need to be improved. The main research on the tube’s dynamic characteristics is to study the tube’s natural frequencies, which is limited by huge equipment and uncertain risky environment under the coal seam, so that some original experiences and conclusions are not applicable to the calculation of tube’s natural frequencies. Furthermore, it is difficult to meet the design requirements and operating parameters of tubes by copying the previous formula studies, and improper operations may even affect the safety of production. Thence, it is significant to have a preliminary theoretical calculation or experimental verification of tube’s natural frequencies before transportation so as to provide a reference for the subsequent design.

This paper uses experiments to verify the theoretical results of the partially liquid-filled tube. Based on the partially liquid-filled tube model, the dynamic characteristics of the tube are experimentally tested, and a method of calculating the natural frequencies by adding the fluid mass to outer tubes is obtained. Moreover, added mass coefficient can be calculated in different conditions. The accuracy is verified by comparing experimental data with numerical simulation results and provides valuable data to the actual drillstring design. The symbols involved in this paper are described in Table 1.

**Table 1:** The description of symbols.

Symbol	Definition
*E*	Modulus of elasticity (Pa)
*I*_*inner*_	Area's moment of inertia for inner tube (m^4^)
*I*_*outer*_	Area's moment of inertia for outer tube (m^4^)
*m*_*inner*_	Mass for inner tube (kg/m)
*m*_*a*_	Added mass of liquid (kg/m)
*m*_*L*_	Mass for inner tube (kg/m)
*m*_*outer*_	Mass for outer tube (kg/m)
*β*	Constant
*ω*	Natural frequencies for empty outer tube (Hz)
*ω*_*L*_	Natural frequencies for liquid-filled outer tube (Hz)
*α*_*inner*_	Added mass coefficient for inner tube
*α*_*outer*_	Added mass coefficient for outer tube
*γ*	Ratio of natural frequencies, *ω*/*ω*_*L*_
*ρ*	Density of PVC-U tube (kg/m^3^)

## Materials and methods

### Mathematical modeling

**Fig 1:**
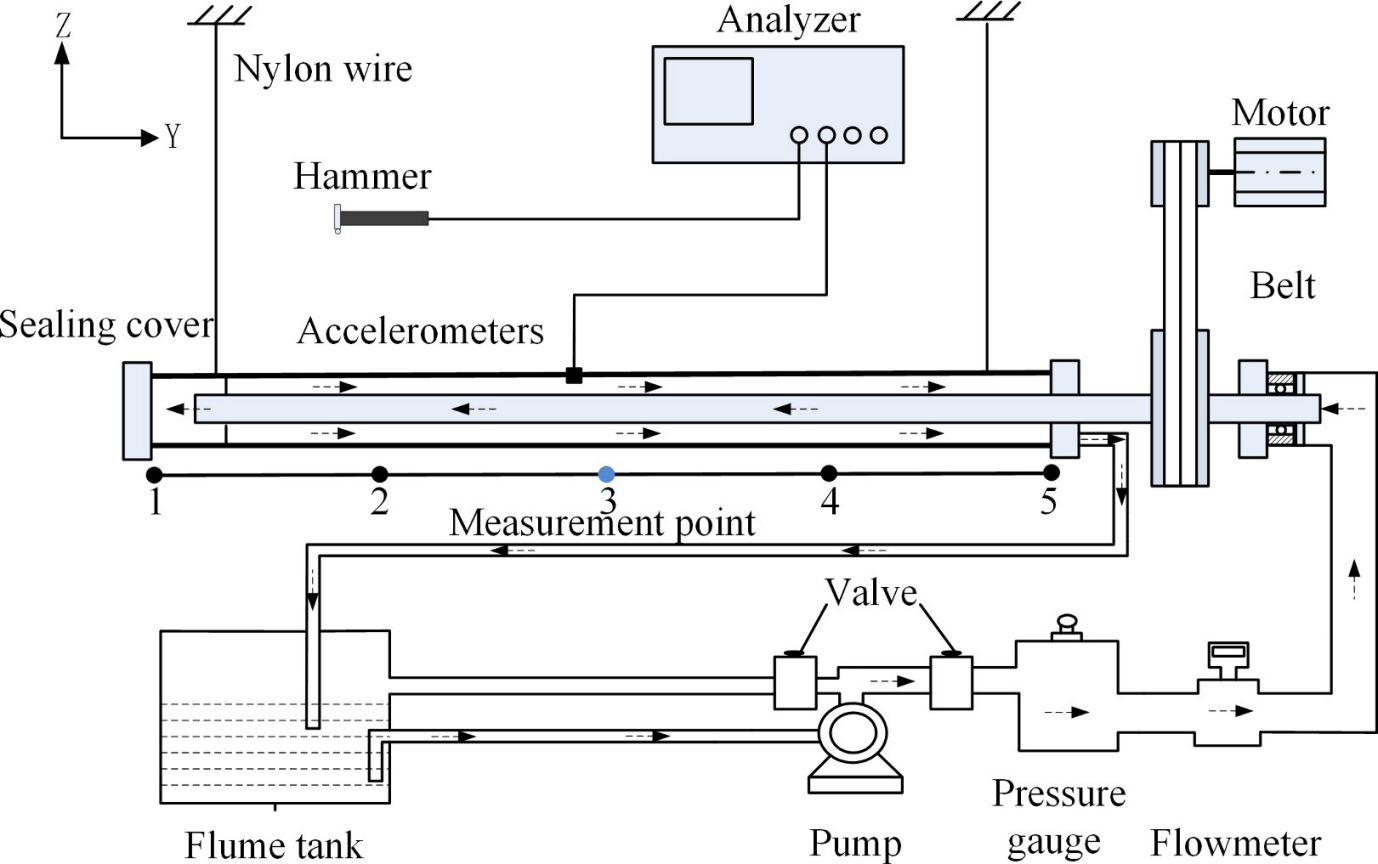
Experimental scheme.

Based on the Euler-Bernoulli beam model [8], Eq (1) and Eq (2) are used to describe the vibration of the inner tube and outer tube of annulus tube which is partially filled with liquid:

$$EI_{inner}\frac{\partial^{4}y_{inner}(x,t)}{\partial x^{4}}+\left(m_{inner}+\alpha_{inner}m_{L}\right)\frac{\partial^{2}y_{inner}(x,t)}{\partial t^{2}}=0$$ (1)

$$EI_{outer}\frac{\partial^{4}y_{outer}(x,t)}{\partial x^{4}}+\left(m_{outer}+\alpha_{outer}m_{L}\right)\frac{\partial^{2}y_{outer}(x,t)}{\partial t^{2}}=0$$ (2)

where *E* is the modulus of elasticity of the annulus tube material and *I* is the area's moment of inertia about the neutral axis of the tube's cross-section. The connection between inner tube and outer tube is a right cap shown in Fig 1 in the next section. It is obvious that the vibration of the inner tube does not directly act on the outer tube, which means it is reasonable to consider Eqs (1) and (2) decoupled. And the fluid used in this paper is water and its viscosity is 1.01×10^-3^Pa·s when the temperature is 293.15K。

The mass for per unit length of the inner tube, the outer tube and the liquid are, respectively, *m*_*inner*_, *m*_*outer*_ and *m*_*L*_. The added mass coefficient of liquid on inner tube is *α*_*inner*_, and the added mass coefficient of liquid on outer tubes *α*_*outer*_ is defined as the ratio between the added mass of liquid *m*_*a*_ and the mass of liquid *m*_*L*_ [9]:

$$\alpha_{outer}=\frac{m_{a}}{m_{L}}$$ (3)

If the fluid is inviscid, *m*_*a*_ is equal to the displaced mass. And the natural frequency *ω*_*L*_ of outer tubes filled with liquid can be directly calculated from the formula:

$$\omega_{L}=\beta^{2}\sqrt{\frac{EI_{outer}}{m_{outer}+\alpha_{outer}m_{L}}}$$ (4)

where *β* is a constant that depends on the vibration mode and the tube's test condition. Eq (4) is valid for small amplitudes of tube vibration.

As mentioned above, the added mass coefficient of outer tubes *α*_*outer*_ can be calculated when the natural frequencies of the outer tubes are obtained. In Eq (4), many variables are involved, which will inevitably affect the result. In order to reduce the deviations caused by parameters *β* related to the properties of the outer tube, the ratio of the natural frequency of the outer tube without liquid *ω* and the natural frequency of the partially filled outer tube is obtained: In order to reduce the impact of parameter *β* on the properties of the outer tube, the ratio of the natural frequencies is obtained:

$$\frac{\omega }{\omega_{L}}=\sqrt{\frac{m_{outer}+\alpha_{outer}m_{L}}{m_{outer}}}$$ (5)

Therefore, the added mass coefficient of outer tubes can be calculated by the experimental data:

$$\alpha_{outer}=\frac{m_{outer}\left(\gamma^{2}-1\right)}{m_{L}}$$ (6)

where $\gamma =\frac{\omega }{\omega_{L}}$.

Therefore, from Eq (6), in order to calculate the added mass coefficient of the liquid in the partially filled outer tubes, it is enough to measure the natural frequencies of the outer tubes under various conditions.

### Experimental processes

The main purpose of this experiment is to obtain the natural frequencies of outer tubes under the condition of the change of tube parameter (outer tube length) and the change of external parameters (eccentricity, fluid velocity, volume fraction). In every test of different parameter, the increasing in the length of the tubes are used. And then the experimental results are verified by numerical simulation. Since it is impossible to measure the natural frequencies of liquid-filled tubes during drilling process, an effective method of calculating the natural frequencies of outer tubes need to be presented, and the natural frequencies will be found by adding the liquid mass to outer tubes mass. In this experiment, PVC-U (*ρ* = 1350kg/m^3^) tubes are used. The information of masses per unit length of the tubes are shown in Table 2. Therefore, the added mass of liquid becomes a significant part of the total mass, it can increase the effect of the liquid on the natural frequencies of the outer tubes. Moreover, the experimental results can be obtained more clearly with the measurement.

**Table 2:** Tube parameters.

Tube No.	1#I	1#O	2#I	2#O	3#I	3#O
Outer diameter (mm)	25	40	25	40	25	40
Inner diameter (mm)	23	37	23	37	23	37
Length (mm)	1000	1000	1500	1500	2000	2000
Mass(kg/m)	0.102	0.245	0.102	0.245	0.102	0.245

According to Kong X, the velocity of water is 3.6 m^3^/h [10]. As shown in Table 2, when the inner diameter of inner tube is 23mm, it can be easily calculated that the velocity of water is almost equal to 2.4m/s. In this paper, the initial inlet is set as the speed inlet and the range of liquid inlet velocity is 0-3m/s. In Fig 1, the right side of outer tube is selected as the outflow test condition to set the normal gradient for all flow parameters except pressure to zero [11]. For different requirements, the inner tube has no stick-slip, and the range of inner tube rotating speed is 0–50 rpm [12].

During the experiment, one end of the PVC-U tube is closed with a sealing cover and the other end is a self-priming pump with the power of *P* = 0.675kW to circulate water inside the annulus tube. The belt is rotated by motor and drive the inner tube’s rotation. The two ends of the tube use two nylon rope to straighten the level [13]. As for Fig 1, the whole experimental facility is a closed liquid circulation system which is composed of annular tubes, flume tanks, pump, pressure gauge and flowmeter. The most obvious advantage of the closed liquid circulation system is that the levels of liquid in the outer tubes can be estimated by the flow rate of pump, the tube's diameter and the storage of the flume tank. The experimental parameters for the PVC-U tubes are shown in Table 2. There are three groups of annular tubes in the experiment. Among them, 1#I and 1#O are the first group, 2#I and 2#O are the second group, and 3#I and 3#O are the third group. In addition, 1#I, 2#I, 3#I tubes are inner tubes, 1#O, 2#O, 3#O are outer tubes.

As shown in Fig 1, the specific experimental process is as follows. (i) According to the actual experiment process, moving the hammer to tap equal spacing points. (ii) At the corresponding measuring point (point 3), a light-weighted high-sensitivity accelerometer is used to measure the vibration responses of the tube. Through the impact hammer (type8206, accuracy of 22.7mV/N) produced by Brüel & Kjær, the incentive system mainly provides external force to the outer tubes. Also, the vibration response system is mainly composed of the ICP accelerometer (type 4508, accuracy of 10mv/(m·s^-2^)) and a USB4431 data acquisition card. (iii) The signal from the impact hammer and accelerometer is collected by a data acquisition card to obtain Frequency Response Functions (*FRF*) of the tube. (iv) The obtained *FRF* is imported into a modal analysis software (Me’scope V5) for data post-processing analysis to obtain the natural frequencies and mode shape under the test.

## Results

**Fig 2:**
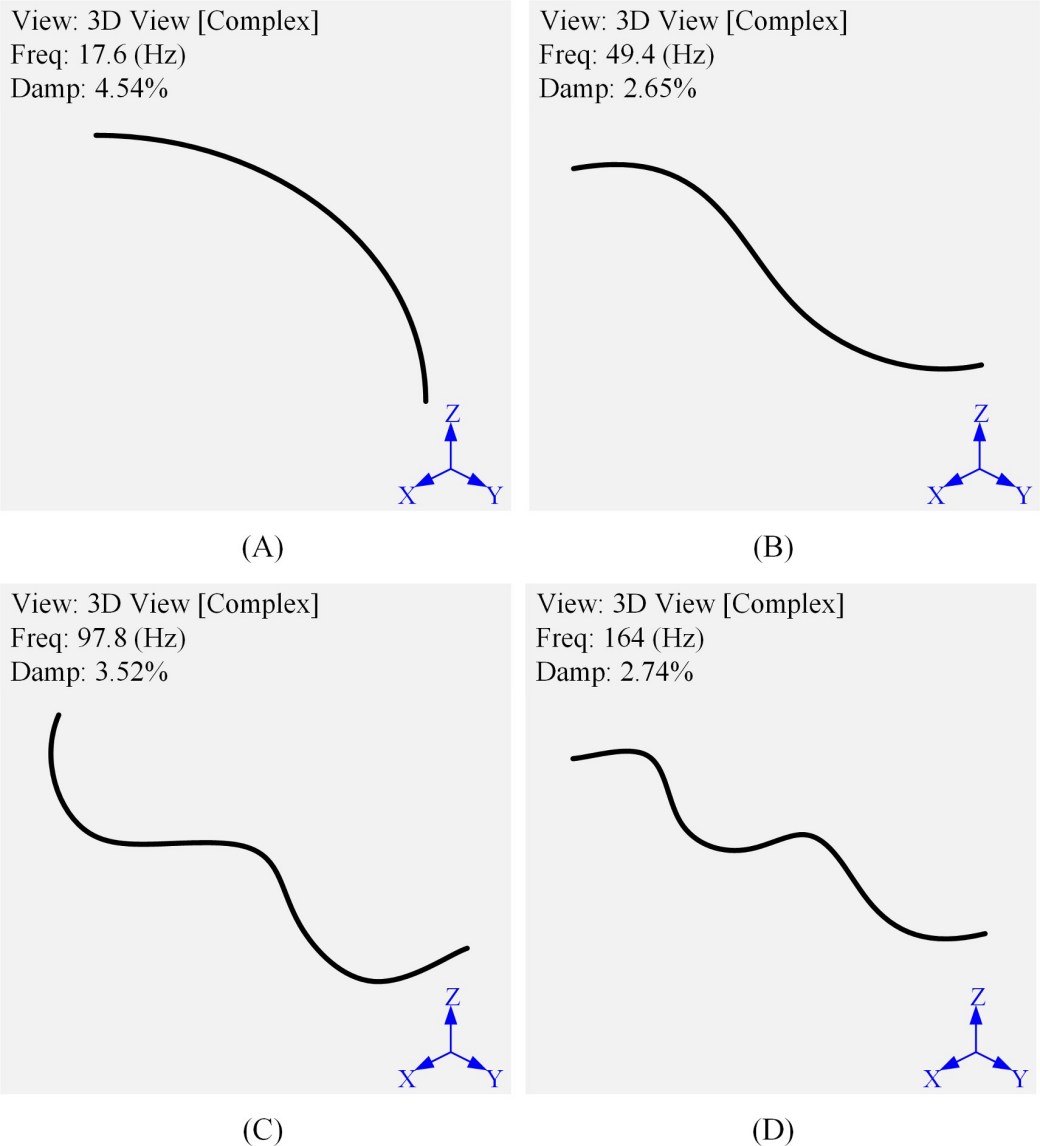
The first four modes of the 3#O outer tube. (A) The first mode. (B) The second mode. (C) The third mode. (D) The fourth mode.

In order to obtain the natural characteristics of outer tubes and compare the change of natural characteristics under different external conditions, the experiment tests the modal on 3#O outer tube to obtain the modes at the first four natural frequencies, the results are shown in Fig 2. From Fig 2, it can be found that the natural frequency of each step of the outer tube and its mode shapes coincide with the analytical results. As it will be discussed inmodal simulation, Finite Element Method (*FEM*) analysis also support this conclusion. Comparing to the later simulation results in modal simulation section, mode shapes of 3#O outer tube obtained by hammer impact tests are similar to simulation results, which verifies the accuracy of experimental results.

### Effect of eccentricity on tube’s natural frequencies

Tube’s eccentricity plays a predominant role in drilling and cementing operations, several investigations focused on the effect of the eccentricity on non-Newtonian fluid flow in annulus tube. However, the effect of eccentricity on tube’s natural frequencies has not been studied. Due to the weight of the drill string, the deviation of wellbore orbit and the distribution of forces on annulus tubes, the annular tube would be eccentric. If the outer tube is eccentric in borehole, the annulus channel would be divided into wide-gap channel and narrow-gap channel, and the eccentricity will affect the fluid transport within the drill string. Therefore, it is necessary to consider the influence of eccentricity on the dynamic characteristics of outer tubes during the actual drilling process.

**Fig 3:**
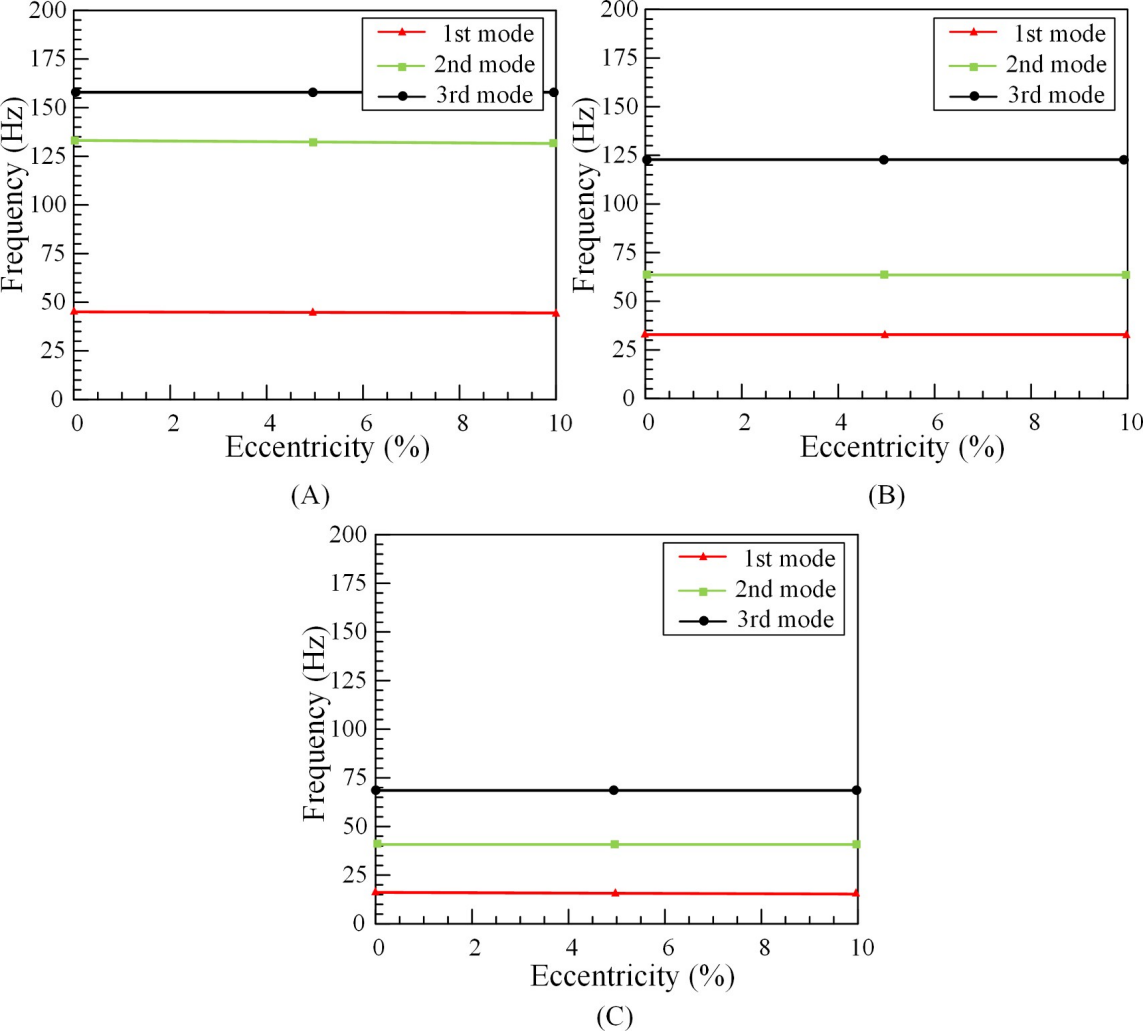
The effect of eccentricity on outer tube’s natural frequencies. (A) 1#O. (B) 2#O. (C) 3#O.

The condition of natural frequencies test is to set the fluid velocity to 1.5m/s, the annulus tubes are fully filled and the rotation speed of inner tubes is 30rpm. According to the experimental results, the first three natural frequencies of the outer tubes are obtained under different tube lengths and degrees of eccentricity. Fig 3 illustrates that eccentricity does not affect the natural frequencies of the tube. When the eccentricity of 1#O, 2#O, 3#O is same at 10%, the first-mode natural frequencies are, respectively, 46 Hz, 33.4 Hz, 15.6 Hz. Compared with the first-mode natural frequency of the 1#O, the first-mode natural frequency of the 2#O is decreased by 27.4%, and the first-mode natural frequency of the tube 3 is even decreased by 66%. In addition, the mass and stiffness of outer tubes are the main factors affecting the outer tube’s natural frequencies, the eccentricity of the fluid does not change the mass and stiffness of the outer tubes. Therefore, in the subsequent experiment and numerical simulation, the factor of eccentricity in the tube will be ignored.

### Effect of fluid velocity on tube’s natural frequencies

In order to study the influence of fluid velocity on the dynamic characteristics of outer tubes, the experiment also uses the self-priming pump with the power of *P* = 0.675KW to pump the water in the tube. The annulus tubes are fully-filled with water and the middle of the annular space is drained to a water circulation system. The test condition of natural frequency test is without eccentricity and the rotation speed of inner tubes is 30rpm. The fluid velocity are, respectively, 0 m/s, 1.5 m/s and 3 m/s.

**Fig 4:**
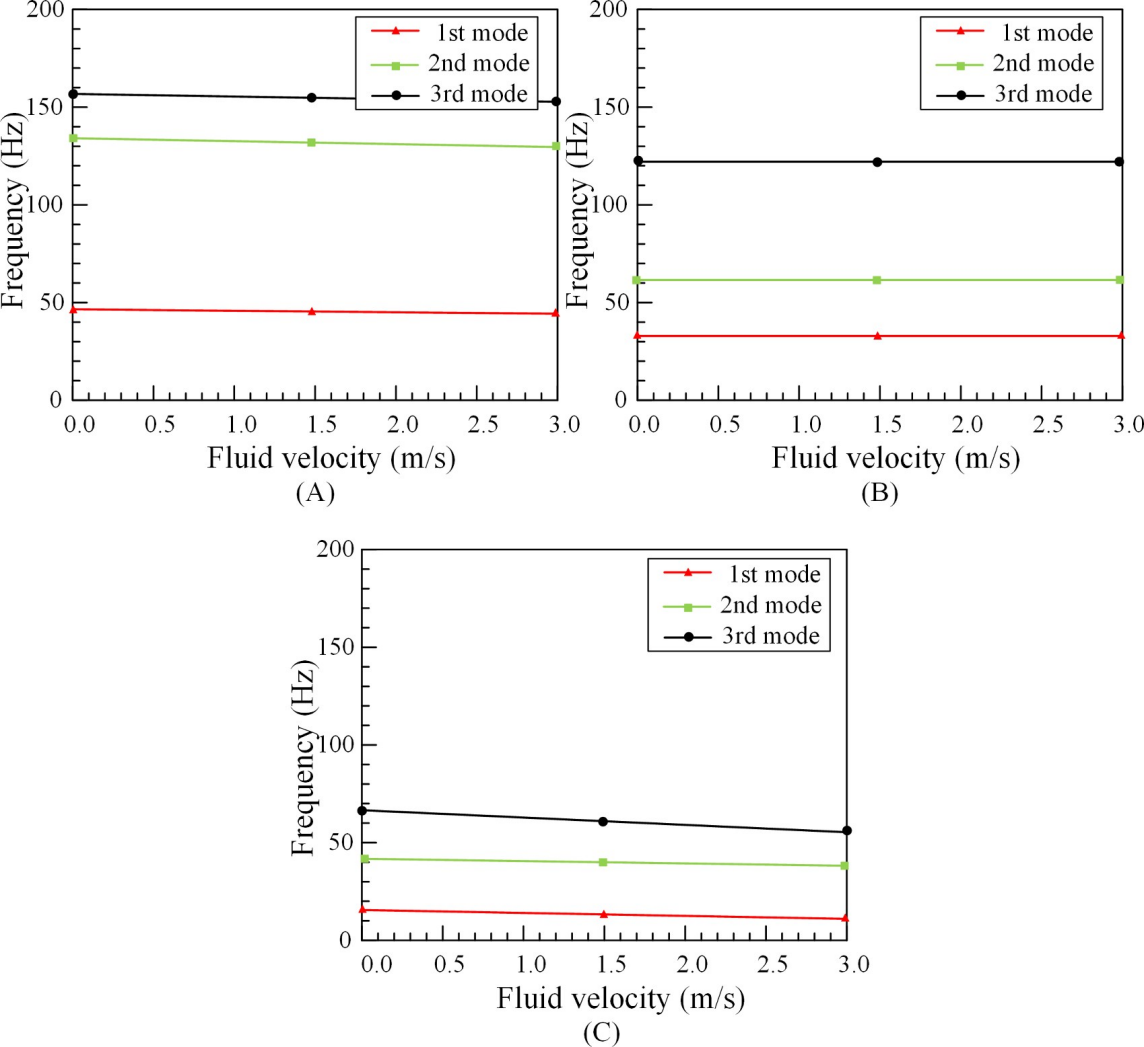
Tube’s natural frequencies under different fluid velocity and lengths. (A)1#O. (B) 2#O. (C) 3#O.

It can be found in Fig 4 that the increase of fluid velocity has little effect on the natural frequencies of outer tubes. The change is basically linear and not obvious. Comparing the influence of the fluid velocity on tube’s natural frequencies under different lengths, it can be found that under the three kinds of lengths, the variation tendencies of these curves are consistent. It is further proved that the effect of fluid velocity can be neglected.

### Effect of outer tube length on tube’s natural frequencies

In order to study the influence of outer tube length on the natural characteristics of outer tubes, regardless the change of fluid mass, modal test on 1#O, 2#O, 3#O tubes without liquid is necessary. The condition of modal test is without eccentricity and the rotation speed of inner tubes is 30rpm.

**Fig 5:**
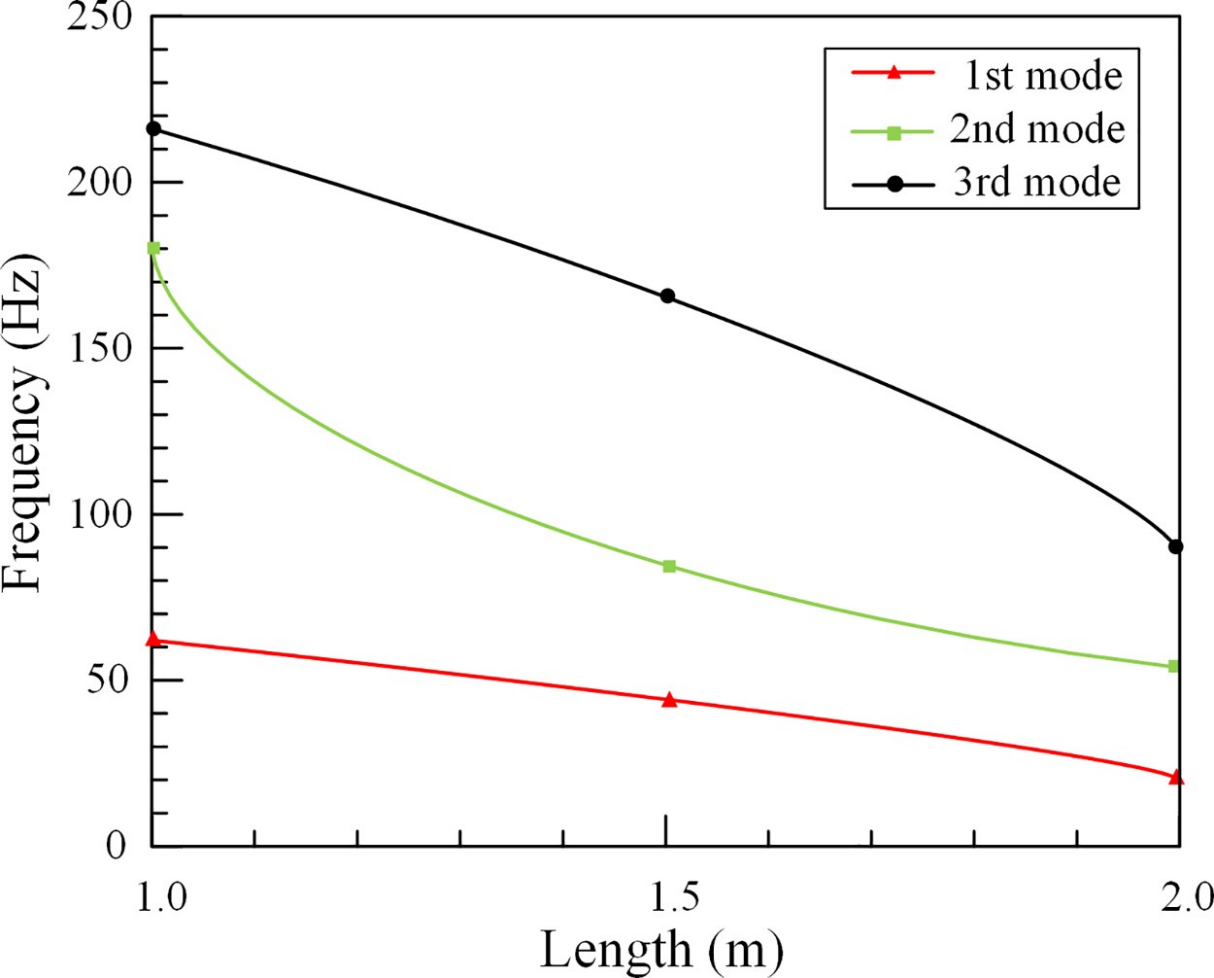
The outer tube’s natural frequencies under different lengths.

According to the experimental results, the first three natural frequencies of outer tubes are obtained under three different lengths. Fig 5 presents that the natural frequencies decrease with the increase of the length of outer tubes. Due to the change of outer tube length, tube’s mass and stiffness change at the same time, and in this case, the change of stiffness is dominant. Also, the calculation of the added mass factor is obtained by testing the natural frequencies of empty outer tubes.

### Effect of volume fraction on tube’s natural frequencies

**Fig 6:**
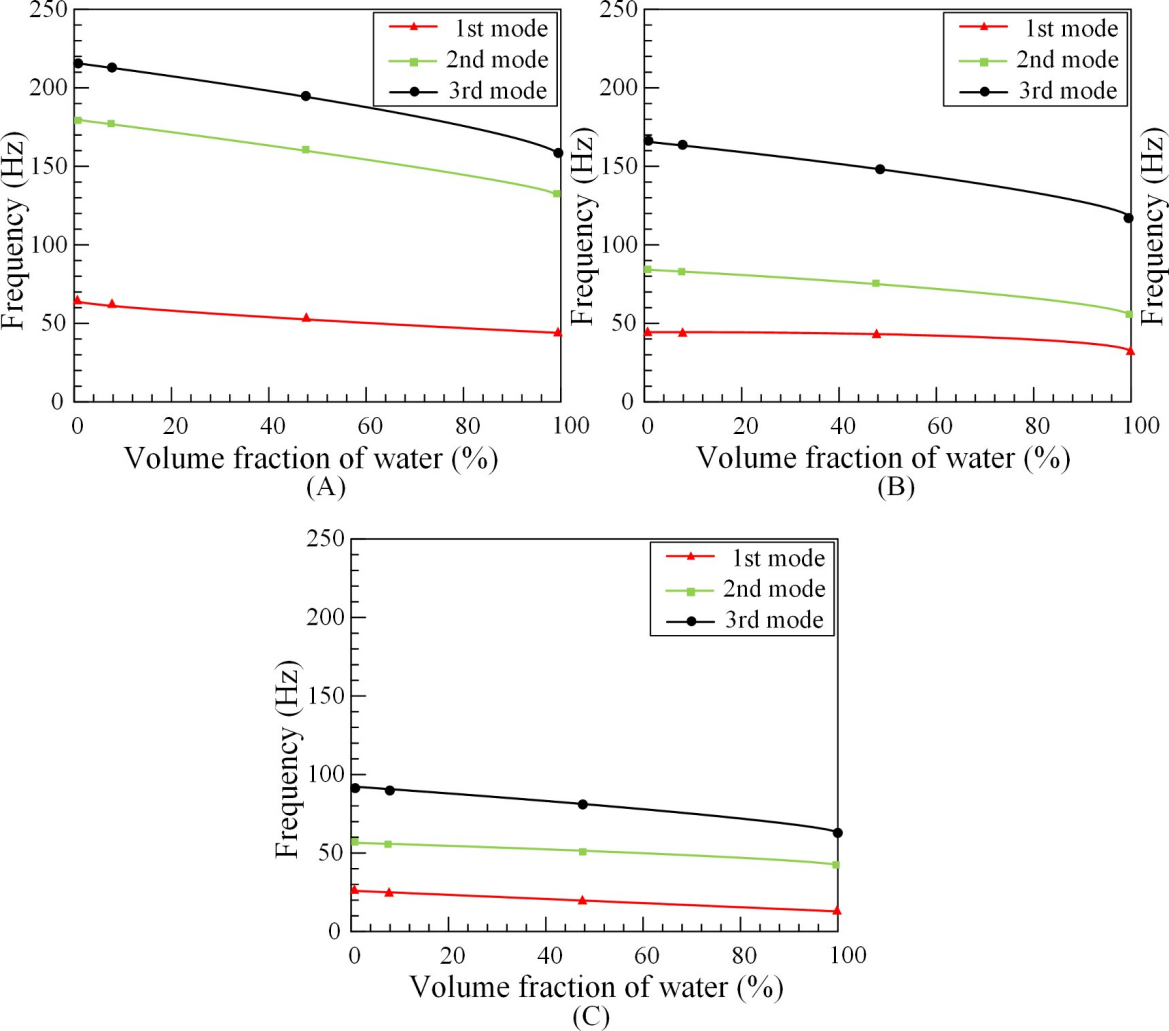
Tube’s natural frequencies under different volume fraction and lengths. (A) 1#O. (B) 2#O. (C) 3#O.

Different filling conditions in annulus tubes would cause changes in natural frequencies of outer tubes. The self-priming pump uses the principle of pressure difference to fill the water stored in flume tank into the annulus tubes. In order to control the volume fraction in annulus tubes, the motor of self-priming pump provides power to this closed liquid circulation system. Condition of modal test is without eccentricity, the rotation speed of inner tubes is 30rpm and the fluid velocity is1.5m/s. The outer tubes are respectively tested in fully-completed, 50% filled, 10% filled and 0% filled with water [8]. Through the modal experiment, the *FRF* curves of the outer tubes under different states are obtained, and the first three natural frequencies are obtained by further analysis. The influence of the volume fraction in outer tubes on the natural frequencies under different lengths is investigated. The analysis results are shown in Fig 6.

Fig 6 exemplifies the change between volume fraction and natural frequencies of outer tubes. With the increase of volume fraction, the natural frequencies of outer tubes decrease. The increase of the volume fraction of the liquid in the tube significantly lower the natural frequencies of the outer tubes. Comparing Fig 6(A)–6(C), it can be seen that the natural frequencies of tube decrease with the increase of volume fraction under different tube lengths. When the tubes of 1#O, 2#O, 3#O are fully-completed, the first-mode natural frequencies are, respectively, 45 Hz, 33 Hz, 15.5 Hz. Compared with the first-mode natural frequency of the 1#O, the first-mode natural frequency of the 2#O is decreased by 26.7%, and the first-mode natural frequency of the tube 3 is even decreased by 65.5%. The shorter outer tube length, the more liquid mass affects the natural frequencies.

### Added mass coefficient

Through the above experimental tests and comparison, it can be found that the natural frequencies of outer tubes have relatively large impact on the volume fraction and the length of tube. However, eccentricity and fluid velocity have little impact on the natural frequencies of tube. Therefore, the length of tube and the change of volume fraction are mainly considered to test the natural frequencies of outer tubes. The change of tube length mainly affects tube stiffness and mass, the natural frequencies of empty outer tubes can be calculated directly from the Eq (4). However, it is very difficult to obtain the change of volume fraction directly by calculation method because the volume fraction is an estimated parameter. Hence, a method of converting the relevant mass of the liquid into the outer tube mass is proposed to directly calculate the natural frequencies of the fully-filled outer tubes.

**Fig 7:**
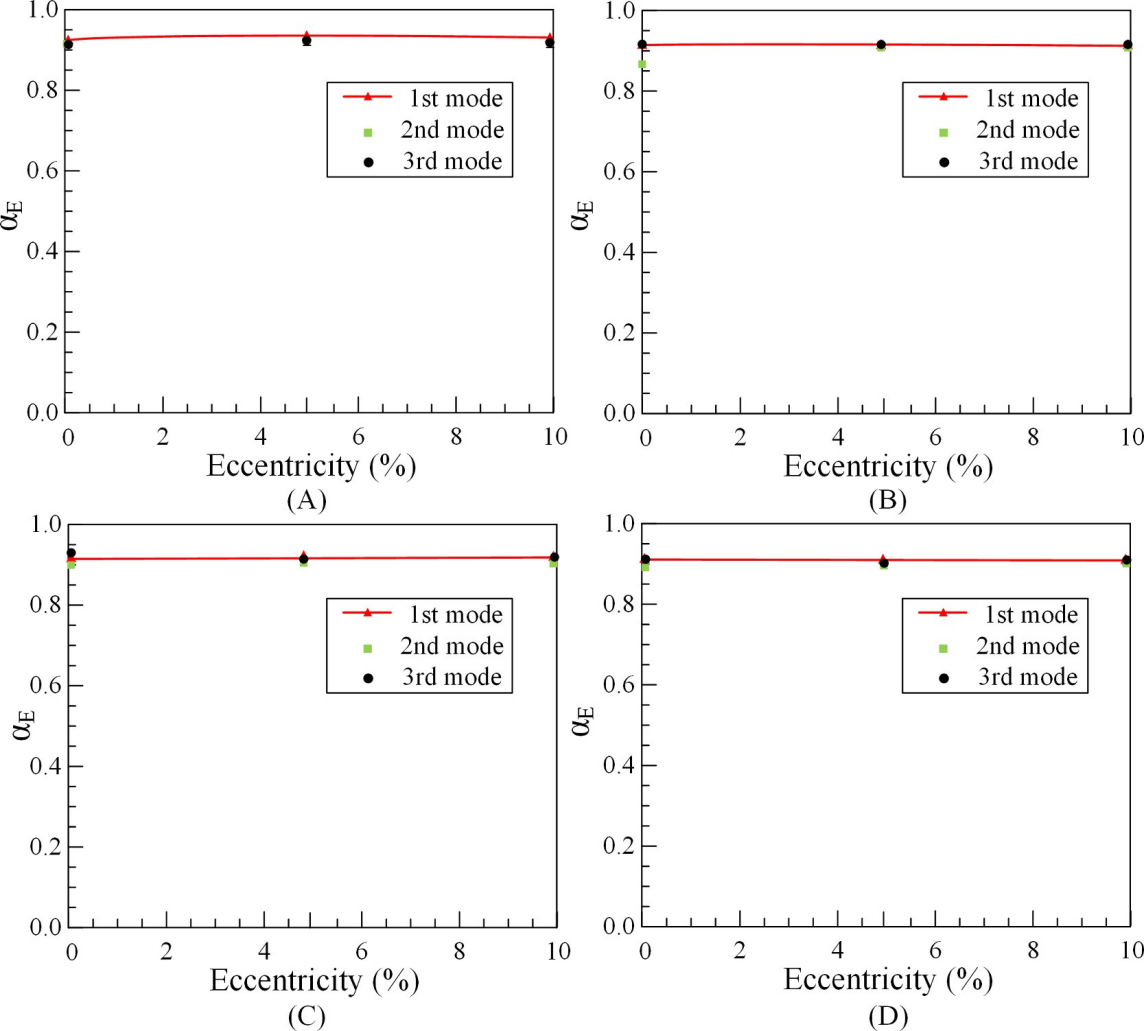
Effect of eccentricity on added mass coefficient of outer tubes. (A) 1#O. (B) 2#O. (C) 3#O. (D) Average *α*_*E*_.

**Fig 8:**
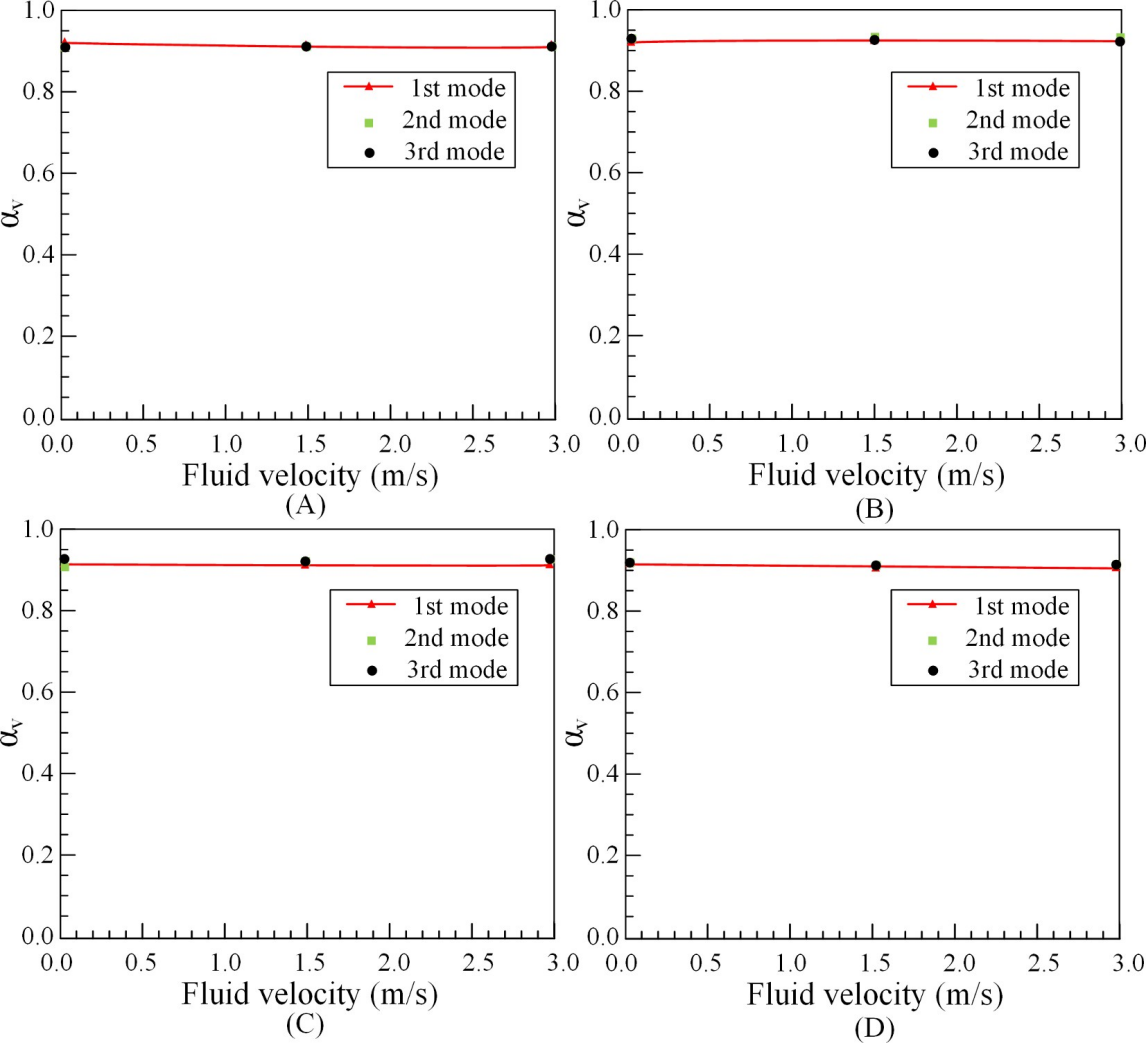
Effect of fluid velocity on added mass coefficient of outer tubes. (A) 1#O. (B) 2#O. (C) 3#O. (D) Average *α*_*v*_.

**Fig 9:**
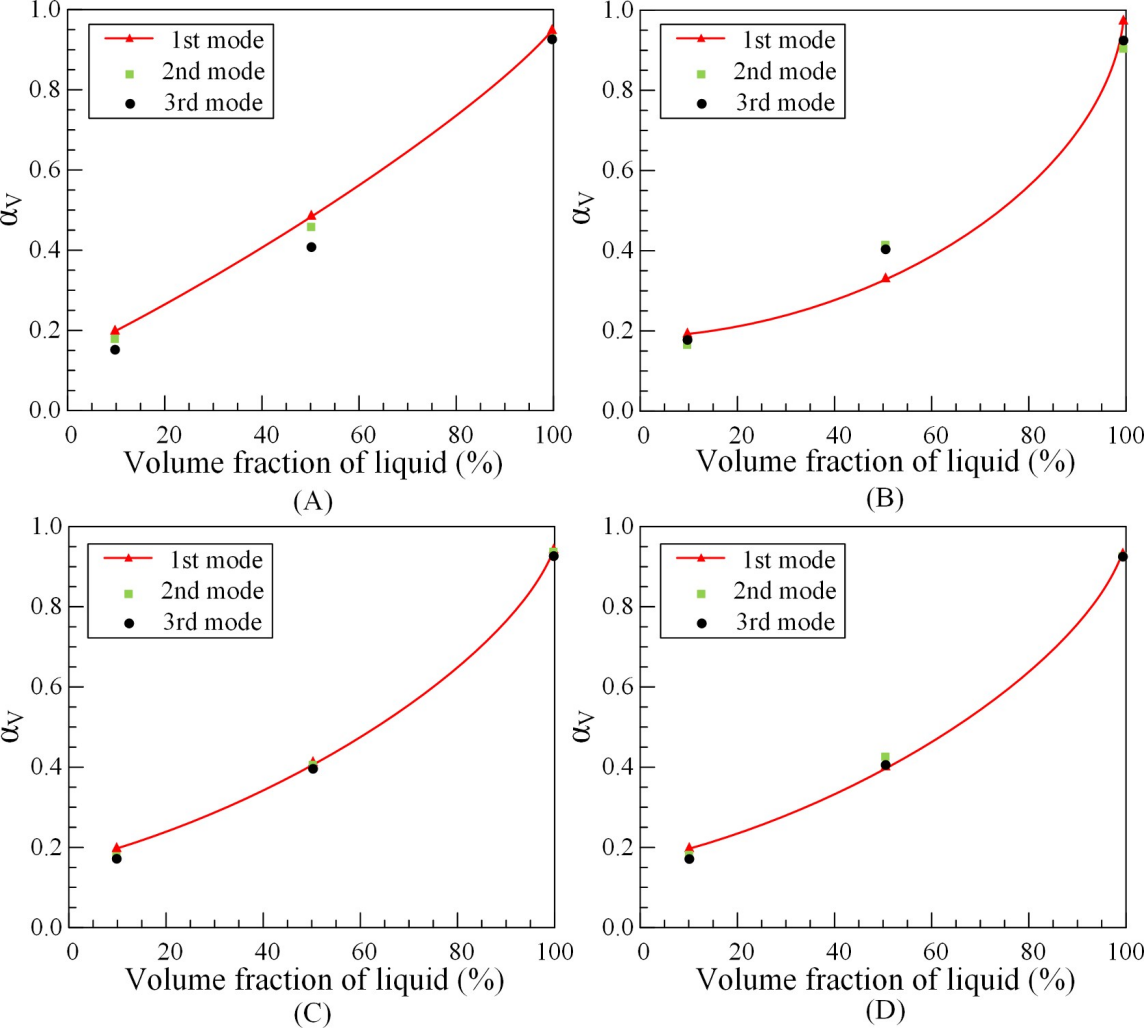
Effect of volume fraction on added mass coefficient of outer tubes. (A) 1#O. (B) 2#O. (C) 3#O. (D) Average *α*_*V*_.

The effects of eccentricity, fluid velocity, outer tube length and volume fraction on the added mass coefficient are investigated. According to Eq (6) and experimental data, the value of the added mass coefficient of outer tubes *α*_*outer*_ for different conditions can be calculated by 1#O, 2#O and 3#O tubes. The added mass coefficient of outer tubes under different eccentricity, fluid velocity and volume friction are, respectively, *α*_*E*_, *α*_*v*_ and *α*_*V*_. The calculation results are shown in Figs 7–9.

Basically, since the eccentricity and flow velocity almost not affect the natural frequencies of outer tube, the add mass coefficient is barely a fixed value. From Figs 7 and 8, when the outer tube is fully filled, approximately 92% of the fluid mass can be added to tube mass. Fig 9 illustrates that the added mass coefficient of partially liquid-filled outer tubes is not a fixed value, which changes with the volume fraction of fluid changes. When volume fraction of fluid in the tube increases, the added mass coefficient *α*_*V*_ also tends to increase. When the outer tube is10% filled with water, the value of the added mass coefficient is nearly 0.2. When the outer tube is half filled, the value of the added mass coefficient is roughly 0.38. However, when the volume fraction of fluid almost reaches 100%, the value of the added mass coefficient is about 0.95. In other words, when the tube is full, the entire volume can be attached to the outer tubes to calculate the natural frequencies.

## Discussion

### Modal simulation

**Fig 10:**
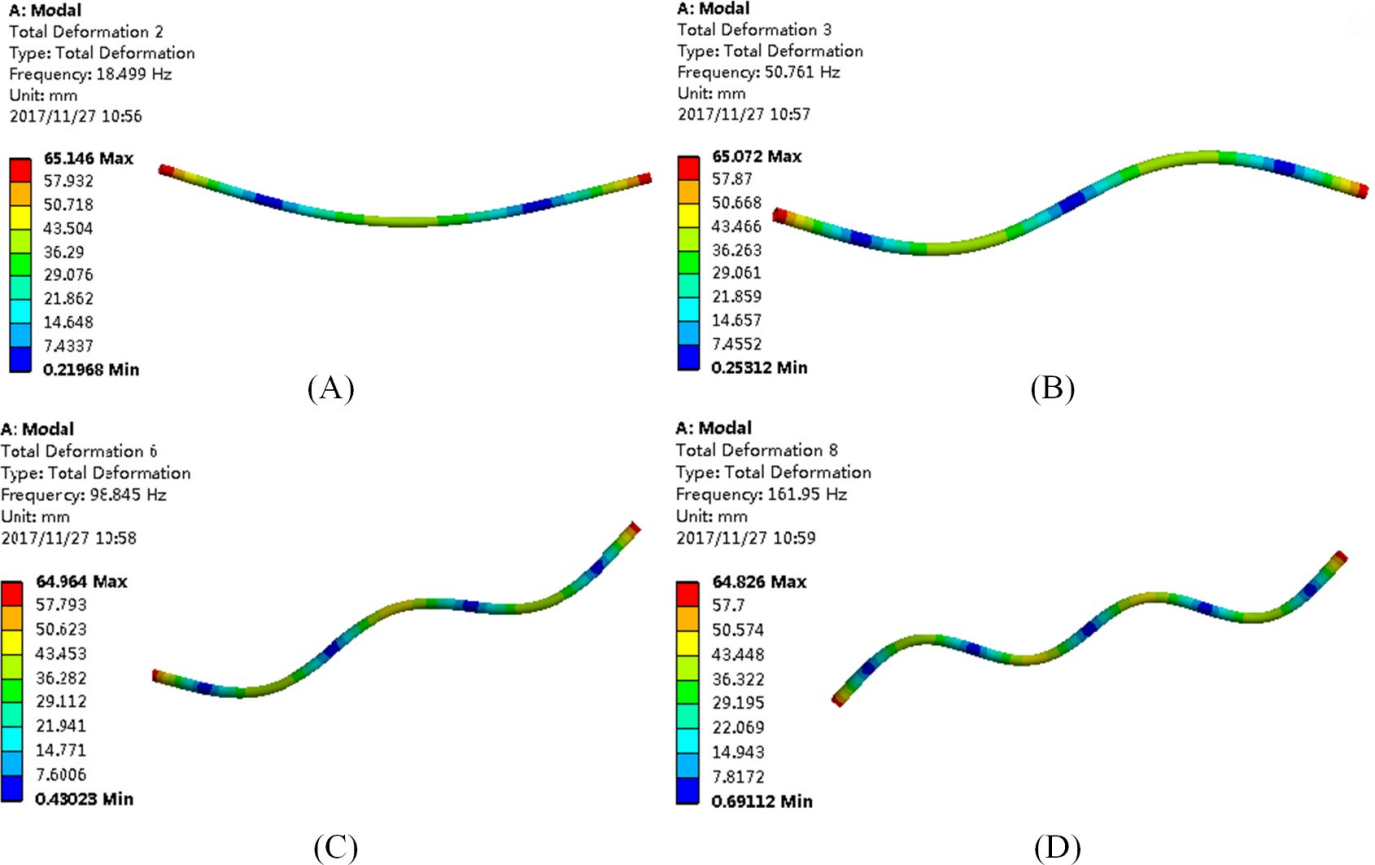
The first four modes of the 3#O tube under numerical simulation. (A) The first mode. (B) The second mode. (C) The third mode. (D) The fourth mode.

In this section, results obtained from experiments and numerical simulations are compared and analyzed. The previous section presents the modal test on the natural characteristics of outer tubes and studies the variation of the tube’s natural frequencies under different external parameters. The added mass coefficient of outer tubes is obtained by calculation. In order to verify the accuracy of experimental data, *FEM* analysis of outer tube’s natural frequencies are conducted in an anhydrous state. The natural frequency and natural mode are obtained by setting the tube parameters and applying modal simulation, the results are shown in Fig 10.

**Fig 11:**
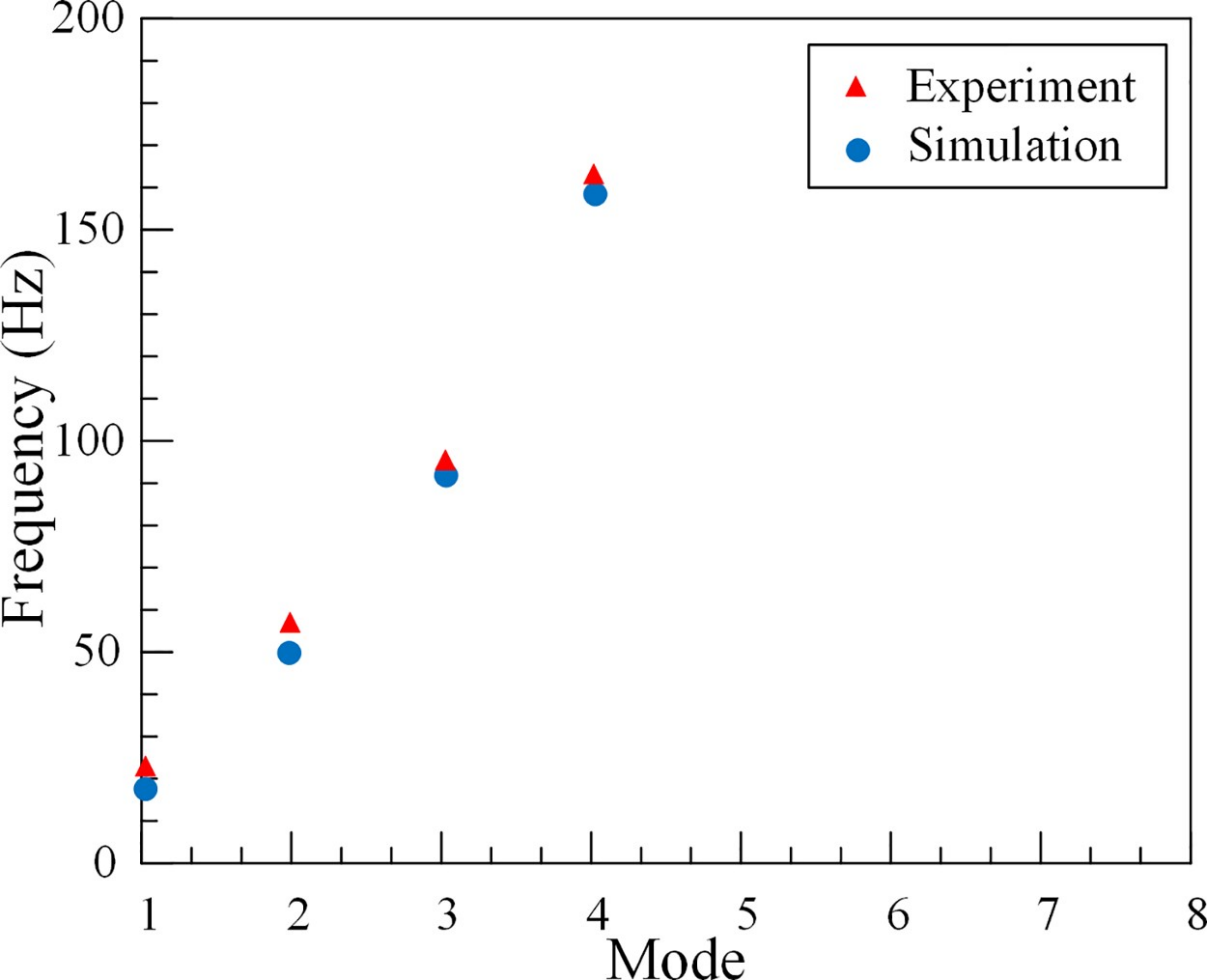
Results comparison in 3#O outer tube’s natural frequencies.

Comparing the experimental and *FEM* results in Fig 11, it can be found that the results of experiment and simulation are almost identical for the waterless tube, which proves that the data measured by the experiment are correct.

### Comparison of experimental and simulation results

**Fig 12:**
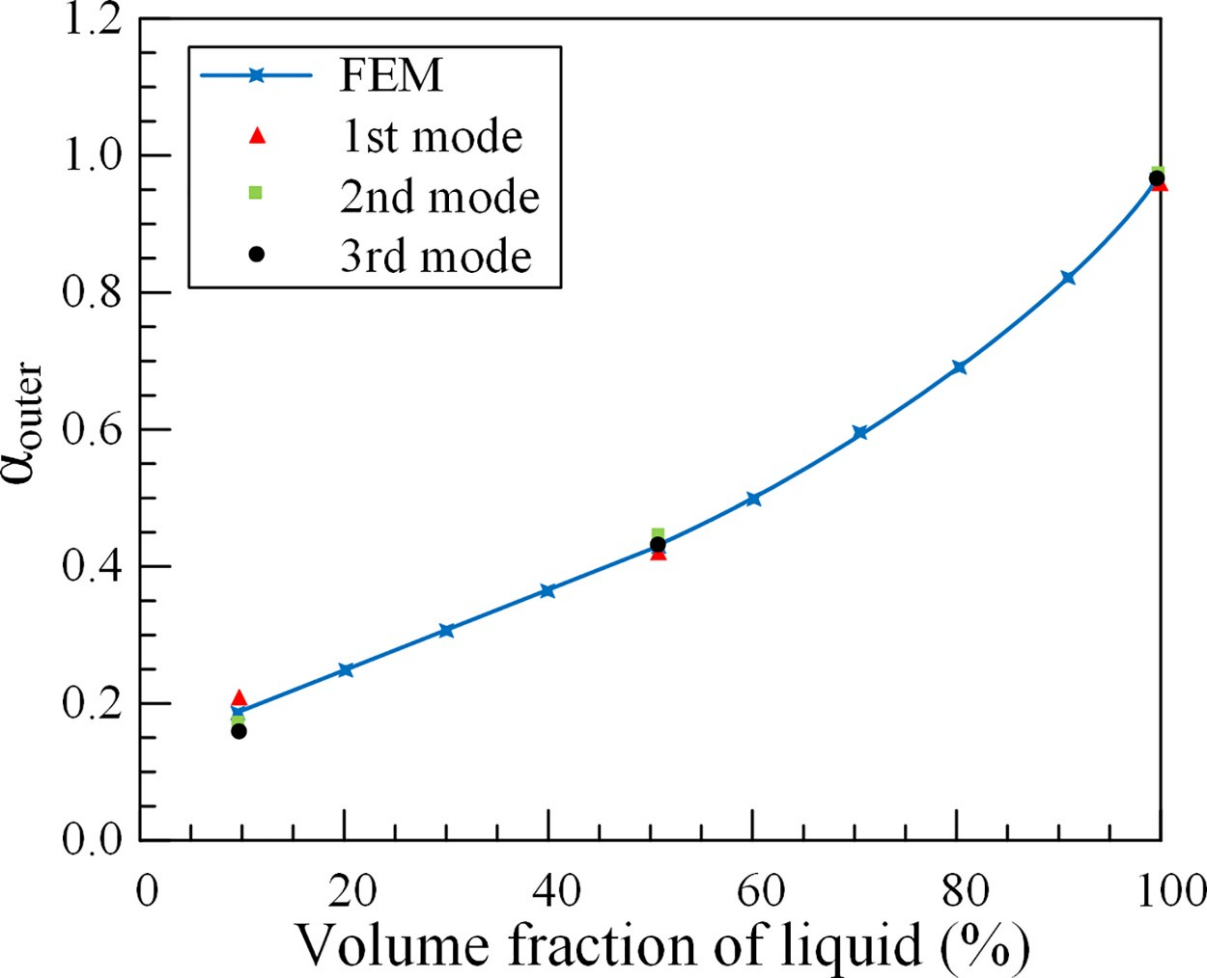
Results comparison in added mass coefficient of 3#O outer tube.

As shown in Fig 12, the added mass coefficient of outer tubes *α*_*outer*_ increases as the volume fraction of liquid increases. The experimental results are in good agreement with the numerical results. Under a fixed volume fraction, the fixed added mass coefficient of outer tubes *α*_*outer*_ for the partially liquid-filled annulus tube can be obtained, which is substituted into the Eq (6) and solved for the natural frequencies.

## Conclusions

In this paper, dynamic characteristics analysis of partially liquid-filled tubes in a drillstring system was investigated through the added mass method. The annulus tubes are used to simulate the fluid transportation process between drilling rod and casing tube when drilling horizontally. And the annular tubes are divided into an inner tube and an outer tube, and both are modeled by the Euler-Bernoulli theory. For partially liquid-filled annulus tubes, the changes of tube’s the natural frequencies under different conditions were studied. And through experimental tests and numerical simulations, the roots of the changes can be determined.

When the outer tube is fully filled, eccentricity and fluid velocity have little effect on the natural frequencies of outer tubes, and approximately 0.92 of the fluid mass can be added to tube mass. Moreover, the physical parameters of the tube (length) and the volume fraction of the liquid filled in tube have a great influence on the natural characteristics of tube. In order to study the dynamic characteristics of the tube accurately, the added mass method is used and added mass coefficient can be calculated in different conditions. The added mass coefficient of fluid is not a fixed value, it varies with the volume fraction of fluid in the tube. When the outer tube is 10% filled with water, the value of the added mass coefficient is nearly 0.2. As the volume fraction increases to 50%, the value of the added mass coefficient is roughly 0.38. However, when the volume fraction of fluid almost reaches 100%, the value of the added mass coefficient is about 0.95. These results can be used for detecting the dynamic characteristics of partially liquid-filled tubes to avoid strong vibration and ensure the safety of tube transportation during drilling.
